# Supplementary material for: 3D Culture Systems for Exploring Cancer Immunology
Source: Cancers (Basel). 2020 Dec 28;13(1):56. doi: 10.3390/cancers13010056 (PMC7795162; doi:10.3390/cancers13010056)
Supplement: Supplementary file 1 [file cancers-13-00056-s001.pdf]

Supplementary Table S1. Summary of references categorized by 3D culture system used and field of cancer immunology investigated.

**Spheroids**		
Immunotherapy Penetrance		
Reference #	Authors	Article Title
35	Liu et al.	MALDI-MSI of Immunotherapy: Mapping the EGFR-Targeting Antibody Cetuximab in 3D Colon-Cancer Cell Cultures.
36	Rodallec et al.	From 3D spheroids to tumor bearing mice: efficacy and distribution studies of trastuzumab-docetaxel immunoliposome in breast cancer.
Lymphocyte Infiltration		
38	Mark et al.	Cryopreservation impairs 3-D migration and cytotoxicity of natural killer cells.
39	Crourau et al.	Cocultures of human colorectal tumor spheroids with immune cells reveal the therapeutic potential of MICA/B and NKG2A targeting for cancer treatment
Tumor Dormancy/Immunosuppression		
44	Liu et al.	Blockade of IDO-kynurenine-AhR metabolic circuitry abrogates IFN-γ-induced immunologic dormancy of tumor-repopulating cells.
45	Stüber et al.	Inhibition of TGF- β- Receptor signaling augments the antitumor function of ROR1-specific CAR T-cells against triple-negative breast cancer.
46	Raghavan et al.	Ovarian cancer stem cells and macrophages reciprocally interact through the WNT pathway to promote pro-tumoral and malignant phenotypes in 3D engineered microenvironments.
47	Kuen et al.	Pancreatic cancer cell/fibroblast co-culture induces M2 like macrophages that influence therapeutic response in a 3D model.
48	Chandrakesan et al.	DCLK1-Isoform2 Alternative Splice Variant Promotes Pancreatic Tumor Immunosuppressive M2-Macrophage Polarization.
58	Rocha et al.	3D Cellular Architecture Affects MicroRNA and Protein Cargo of Extracellular Vesicles.
59	Thippabhotla et al.	3D cell culture stimulates the secretion of in vivo like extracellular vesicles.
60	Muller et al.	Human tumor-derived exosomes (TEX) regulate Treg functions via cell surface signaling rather than uptake mechanisms.
**Organoids**		
Antigen Presentation		
61	Chakrabarti et al.	Hedgehog signaling induces PD-L1 expression and tumor cell proliferation in gastric cancer.
62	Dijkstra et al	Generation of Tumor-Reactive T Cells by Co-culture of Peripheral Blood Lymphocytes and Tumor Organoids.
Fibroblast-Cancer Interactions		
65	Tsai et al.	Development of primary human pancreatic cancer organoids, matched stromal and immune cells and 3D tumor microenvironment models.
66	Rhee et al.	Fibroblasts in three dimensional matrices: Cell migration and matrix remodeling.

67	Hanley et al.	Tumor resident stromal cells promote breast cancer invasion through regulation of the basal phenotype.
68	Oszvald et al.	Fibroblast-Derived Extracellular Vesicles Induce Colorectal Cancer Progression by Transmitting Amphiregulin.
69	Liu et al.	Cancer-Associated Fibroblasts Provide a Stromal Niche for Liver Cancer Organoids That Confers Trophic Effects and Therapy Resistance.
CAR Cell Development		
18	Dillard et al.	Colorectal cysts as a validating tool for CAR therapy.
72	Leuci et al.	CSPG4-Specific CAR.CIK Lymphocytes as a Novel Therapy for the Treatment of Multiple Soft-Tissue Sarcoma Histotypes
73	Jacob et al.	A Patient-Derived Glioblastoma Organoid Model and Biobank Recapitulates Inter- and Intra-tumoral Heterogeneity.
75	Schnalzger et al.	3D model for CAR-mediated cytotoxicity using patient-derived colorectal cancer organoids.
Personalized Immunotherapy Testing		
79	Votanopoulos et al.	Model of Patient-Specific Immune-Enhanced Organoids for Immunotherapy Screening: Feasibility Study.
80	Neal et al.	Organoid Modeling of the Tumor Immune Microenvironment.
**Microfluidic Chips**		
Immune Checkpoints		
81	Jenkins et al.	Ex vivo profiling of PD-1 blockade using organotypic tumor spheroids.
84	Diskin et al.	PD-L1 engagement on T cells promotes self-tolerance and suppression of neighboring macrophages and effector T cells in cancer.
85	Schmidt et al.	Assessment of Clinical Activity of PD-1 Checkpoint Inhibitor Combination Therapies Reported in Clinical Trials.
Migration, Extravasation and Angiogenesis		
88	Mascolo et al.	Nanoformulated Zoledronic Acid Boosts the Vδ2 T Cell Immunotherapeutic Potential in Colorectal Cancer.
89	Aung et al.	An Engineered Tumor-on-a-Chip Device with Breast Cancer–Immune Cell Interactions for Assessing T-cell Recruitment.
90	Cui et al.	Hacking macrophage-associated immunosuppression for regulating glioblastoma angiogenesis.
